# The Addis Health and Demographic Surveillance System (Addis-HDSS): Context and Methods

**DOI:** 10.4314/ejhs.v34i2.2S

**Published:** 2024-12

**Authors:** Semira Abdelmenan, Hanna Yemane Berhane, Sitota Tsegaye, Nebiyou Fasil, Dagmawit Tewahido, Tigest Shifraw, Firehiwot Workneh, Walelegn Worku, Yoseph Yemane Berhane, Dongqing Wang, Uttara Partap, Wafaie Fawzi, Meaza Demissie, Alemayehu Worku, Yemane Berhane

**Affiliations:** 1 Department of Epidemiology and Biostatistics, Addis Continental Institute of Public Health; 2 Department of Nutrition and Behavioral Science, Addis Continental Institute of Public Health; 3 Department of Global Health and Health Policy, Addis Continental Institute of Public Health; 4 Department of Reproductive Health and Population, Addis Continental Institute of Public Health; 5 Department of Global and Community Health, College of Public Health, George Mason University, Fairfax, Virginia, United States of America; 6 Department of Global Health and Population, Harvard T.H. Chan School of Public Health, Harvard University, Boston, Massachusetts, United States of America

**Keywords:** Addis-HDSS, Health and Demographic Surveillance System, Addis Ababa, Ethiopia

## Abstract

**Background:**

Accurate population-based data is essential for evidence-based public health decision-making. Monitoring health events and evaluating interventions through a population-based platform enables timely decisions in rapidly growing urban areas. Such platforms are rare in African countries. This paper outlines the procedures for establishing the Addis Health and Demographic Surveillance System (Addis-HDSS).

**Methods:**

The Addis-HDSS, located in Yeka sub-city of Addis Ababa, Ethiopia, conducted its first census from December 3, 2022, to January 18, 2023. Each enumeration area was identified and mapped digitally. All households in the study area were visited and geocoded. The baseline census gathered data on sociodemographic status, housing conditions, economic status, and selected health-related factors.

**Results:**

A total of 30,533 households and a population of 107,494 were recorded. The response rate was 99.1%, reflecting high community engagement The average household size was 3.5, and the sex ratio was 81 males to 100 females. The population structure resembled a typical low-income country profile.

**Conclusion:**

Establishing an urban HDSS was feasible with reasonable effort due to the presence of a digital map and the willingness of the urban population. This surveillance system will be an asset to generate reliable urban health and demographic information by providing an unbiased sampling frame for health-related studies. The HDSS will also be used to test the effectiveness of population-based public health interventions.

## Introduction

Reliable and up-to-date demographic and health data are crucial for planning, implementing, and evaluating health programs, especially in low-income countries (1). Policymakers and health system managers need actionable data to improve healthcare delivery and track progress toward health goals. However, many low-income countries suffer from weak or fragmented data systems that fail to provide timely, reliable information (2).

Vital registration systems routinely gather information on vital events to inform population and health policies (3,4). The critical vital events recommended by the United Nations include birth, death, migration, and marriage (5). However, civil registration and vital statistics systems are non-existent or significantly affected by limited resources and systems leading to incomplete data in most low- and middle-income countries (3,4). Therefore, those countries adopted the health and demographic surveillance system to generate reliable and quality data.

A health and demographic surveillance system (HDSS) is a population-based health and vital event registration system that regularly monitors demographic and health events in a geographically defined population (6–8). These events include births, deaths, and migration, and are updated periodically, with intervals ranging from quarterly to biannual to yearly (9). Such a dynamic platform can detect new health events, track long-term population changes through fertility and migration rates, and measure interventions' effectiveness, thereby generating evidence to guide health policy and programmatic decision-making (3,6).

HDSS emerged in the mid-20th century as a response to the need for detailed, continuous mortality and morbidity data in low-income countries (6,9). The earliest HDSS sites include the Gwembe HDSS (Zambia), established in 1956 (10) to study the socioeconomic impact of Lake Kariba on the surrounding communities (11); the Ballabgarh HDSS (India), established in 1961 (12) to demonstrate a model health-care delivery system for rural India; the Niakhar HDSS (Senegal) established in 1962 (13) which hosted vaccine trials that influenced vaccination policies (13–15); and the Matlab HDSS (Bangladesh) established in 1966 (16,17), which was extensively used for evaluating health interventions (16,18). The Butajira HDSS, established in 1986, was the first HDSS in Ethiopia (19,20). Butajira exemplifies the critical role of HDSS in building research capacity in low- and middle-income countries (9). Subsequently, many universities in Ethiopia have established HDSS sites, mainly in rural areas (21). The Harar HDSS established by Haramaya University was the first HDSS in an urban setting in Ethiopia (22). Addis-HDSS is the second urban-only HDSS in Ethiopia.

The need to obtain critical information in urban settings in low-income countries is urgent because of the demographic transition, migration, and rapidly changing lifestyle that affects the health and well-being of citizens. Addis Ababa is one of the rapidly growing cities in Sub-Saharan Africa (23). The city's population grew from less than half a million in 1984 (24) to over 3.5 million in 2007, a sevenfold increase in about two decades, when the last population census was done in Ethiopia. Although no census was done recently to know the exact number, the city population keeps increasing mainly due to the influx of people from other parts of the country. Interpreting health-related statistics has become difficult because of the lack of accurate denominators. Establishing an HDSS in Addis Ababa is thus imperative to generate essential health and demographic information and to host population-based research. The Addis-HDSS was established to generate health and demographic information and to identify population groups for specific studies by offering an unbiased sampling frame in Addis Ababa, Ethiopia. This paper describes the context and methods of the Addis-HDSS.

## Methods

**Figure 1:**
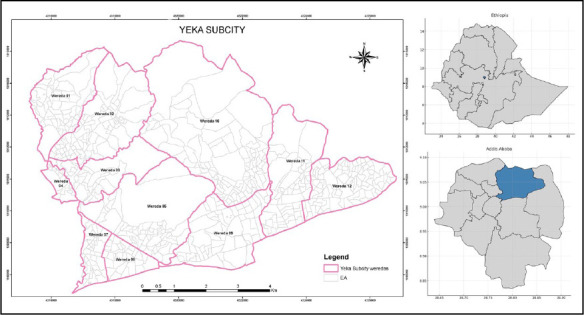
Yeka sub-city, Addis Ababa, Ethiopia

**Study setting and population**: The Addis-HDSS is located in the Yeka Sub-city of Addis Ababa, the capital city of Ethiopia (Figure 1). The sub-city is one of the eleven sub-cities in Addis Ababa and is bordered in the north and northwest by the Oromia region and Gulele sub-city, in the East by the Lemi Kura sub-city, in the south and southwest by Bole and Kirkos sub-cities, and in the south by Arada sub-city. The site was selected through a consultative process with relevant federal, regional, and local stakeholders drawn from government administrative and health agencies, academic institutions, and non-governmental organizations. The factors considered include proximity to the center of the city, demographic diversity of the population, and the relative stability of settlements.

At the initial census, Yeka sub-city had 11 woredas (districts). The area has a total of 668 enumeration areas (sub-locations) based on mapping obtained from the central statistics agency of Ethiopia. The sub-city had two government hospitals, ten health centers, and 84 private health facilities. The sub-city is a mixed-use settlement where residences and businesses are intertwined. New constructions to transform villas into apartment buildings were also commonly observed. Some parts of the sub-city were also earmarked for development projects. Several public and private primary and secondary schools are available in each woreda of the sub-city.

The Addis-HDSS includes six of the 11 districts: Woreda 3, Woreda 4, Woreda 5, Woreda 7, Woreda 8, and Woreda 9. These districts were selected because they were less affected by the city's re-settlement and re-demarcation initiatives. A baseline census was conducted from 03 December 2022 to 18 January 2023.

**Data collection procedure**: The Addis-HDSS initial census covers all houses, households, and populations across 240 enumeration areas. The Central Statistics Authority digitally demarcated the enumeration areas. The digital maps for each enumeration area helped identify and enumerate houses. The field team used the Avanza mapping software (25) to read the digital maps and demarcate the enumeration areas based on the recorded geocoordinates. In addition, local guides help identify landmarks in each enumeration area.

The field teams were trained before the census on research ethics, objectives and importance of HDSS, and data collection protocol, including how to approach households, obtain informed consent, and administer the census tools. The field data collectors and supervisors underwent additional training to understand their responsibilities in managing field teams, ensuring data quality, and accurately demarcating enumeration areas. Additionally, the field teams were trained and standardized on the measurement of mid-upper arm circumference (MUAC). A pretest was conducted outside the Addis-HDSS sites within the Yeka sub-city.

**Measurement**: A house was defined as a structure made from permanent building materials. A household was defined as any group of people living together, sharing food, and recognizing the same household head. A resident was recognized as any person living or intending to stay in a household for at least six months. This definition of resident included students with temporary residence elsewhere but returned for vacation or planned to relocate back after finishing school.

The variables collected at the initial census include socioeconomic, demographic, and selected health-related variables. The data collection questionnaire captured household location, including GPS coordinates, sociodemographic status, economic information, water and sanitation access, healthcare-seeking behaviors, perceived health status, and MUAC measurement (Table 1). Enumerators made three visits before declaring a household a non-responding household.

**Table 1:** Information collected during the baseline census, Addis-HDSS, December 2022-January 2023

Section	Information
House registration	GPS coordinates, household ID, household head
Household	House type, house ownership, water source, sanitary facility, power supply
Individual	Birth date, sex, relationship to the head of the household, marital status, educational status, employment status, physical or mental disability, chronic illness, perceived health status, MUAC
Married women of reproductive age	Current pregnancy status, intention to get pregnant, contraceptive utilization

**Data management and analysis**: Data were collected electronically using the Open Data Kit (ODK) application (26). The digital form was designed with appropriate question types, skip logic, and validation rules. Completed forms were submitted to a central server daily, with immediate feedback provided to the field teams to resolve any discrepancies.

Descriptive statistics were used to analyze the data, and results were presented using flowcharts, graphs, and tables. Stata version 17 (27) was used for data analysis, and R version 4.2.2 (28) was used to generate the population pyramid.

**Ethical approval**: Ethical approval was granted by the Ethics Review Committees of the Addis Continental Institute of Public Health (ACIPH/IRB/003/2022). Written informed consent was obtained from all households after explaining the purpose of the study. A support letter from the Addis Ababa City Administration Health Bureau facilitated the fieldwork.

## Results

The Addis-HDSS covered 240 enumeration areas across six districts. The number of households in each district varied, with the smallest district containing 2,767 households and the largest 7,011. The average number of households per enumeration area was 127 (±36). The population registered in the baseline census totaled 107,494, with 47,987 males and 59,507 females, resulting in a sex ratio of 81 males per 100 females.

**Figure 2:**
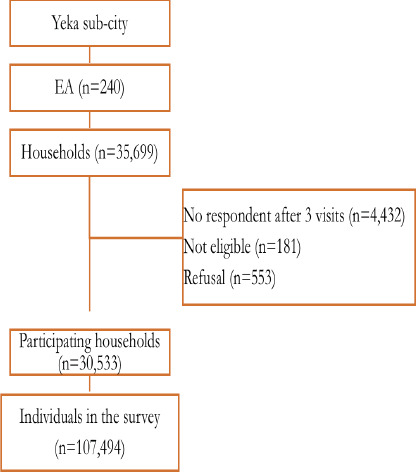
Flowchart of Addis-HDSS baseline survey

Of the 35,699 households visited, 30,533 (85.5%) provided complete information. The remaining 5,166 households were unresponsive after three visits (12.4%), were temporary non-Ethiopian residents (0.5%), or refused participation (1.8%) (Figure 2).

**Figure 3:**
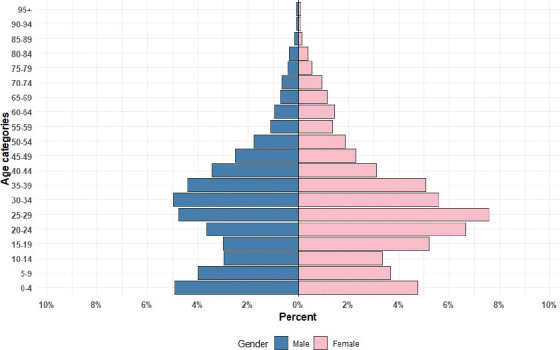
Addis-HDSS population pyramid by age and sex, January 2023

The population pyramid showed a youthful population, particularly in the 20–25 years age group. A dent was observed in the 10-14 and 15-19 age groups (Figure 3). Housing and economic characteristics of the households were as follows: 55.8% of households lived in shared compounds, 27.6% in separate compounds, and 5.2% in government-built condominiums. House ownership varied, with 29.0% owning their houses, while the majority rented from individuals (39.9%) or the government (20.8%). The majority of the houses had rooms designated solely for sleeping (56.5%) and separate rooms for cooking (54.2%). Car ownership was low (10.4%), and electricity access was high (98.3%). Water supply was predominantly within the dwelling (90.7%) (Table 2).

**Table 2:** Economic profile of the households in the Addis-HDSS, Addis Ababa, Ethiopia

Characteristics (N=30, 533)		n (%)
Type of the house	Shared room	61 (0.2%)
	Room in a shared apartment/condominium	260 (0.9%)
	House/room in a shared compound	17,033 (55.8%)
	Government build condominium	1,591 (5.2%)
	Private apartment unit	485 (1.6%)
	House with no compound	2,674 (8.8%)
	House with a separate compound	8,429 (27.6%)
House ownership	Own the house	8867 (29.0%)
	Rented from individuals	12,180 (39.9%)
	Rented from government	6,345 (20.8%)
	Cohabited (no payment)	2,464 (8.1%)
	Other	677 (2.2%)
Houses with room used only for sleeping (yes)		17,264 (56.5%)
Houses with a separate room for cooking (yes)		16,555 (54.2%)
Car ownership (yes)		3,169 (10.4%)
Toilet facility	Water carriage toilet	8,110 (26.6%)
	Improved pit latrine	8,894 (29.1%)
	Traditional pit latrine	11,829 (38.7%)
	No toilet facility	1,700 (5.6%)
Shower facility (yes)		12,976 (42.5%)
Water supply	In own dwelling	27,693 (90.7%)
	Outside dwelling	2,342 (7.7%)
	No access to pipe water	498 (1.6%)
Electric supply (yes)		30,015 (98.3%)
Bank savings	Yes, regularly	2,359 (7.7%)
	Yes, but not regular	13,627 (44.6%)
	Not at all	14,547 (47.7%)

## Discussion

The Addis-HDSS provides a unique opportunity to study a population's health and demographic characteristics in a metropolitan area. The information obtained from such a system can inform public health interventions and policies in similarly rapidly growing big cities in Ethiopia.

The high willingness of the urban population to participate in the Addis-HDSS is a positive indication of their commitment to contributing to evidence generation for public health. The response rate of 99.1% during the baseline census highlights the value that the community places on the importance of evidence generation and community service. This is noteworthy as urban demographic surveillance systems often face challenges regarding community engagement and participation compared to their rural counterparts (29). The willingness of the urban population to engage with the HDSS is crucial for the long-term success and reliability of the data collected. As the Addis-HDSS progresses, one of the primary future directions is conducting longitudinal studies that track changes in the urban population over time. By capturing demographic and health data at regular intervals, the surveillance system can evaluate trends in fertility, mortality, and migration within the urban context, help identify shifts in population dynamics and assess the impact of interventions and policies.

The average EA size (127 ±36) provides valuable insight into the living arrangements in the HDSS sites. Understanding the EA size is essential for sampling in designing future studies, especially intervention studies. The large number of enumeration areas in the Addis-HDSS provides opportunities to use the platform for community-based cluster randomized trials. Each EA has a sufficiently large population and can be regarded as a cluster.

The observed sex ratio of 81 males to 100 females is an interesting demographic feature. Variations in sex ratios can be indicative of factors such as migration patterns or differential mortality rates between sexes. Further analysis and tracking of sex ratios over time may reveal important insights into the population dynamics of the urban setting.

The dent observed in the population pyramid at the age group 10 to 19 years could be influenced by various factors, including lower birth rate in the past, about 10 to 19 years ago. While the broad base shows a high youth population, which also implies a high dependency ratio, as a large portion of the population depends on the working-age population for support and resources.

Using localizing software was critical to demarcate the enumeration areas and identify landmarks documented in the enumeration area maps. The Central Statistical Agency of Ethiopia specifically designed the enumeration area map for the census. It was used to divide the study area into smaller sections or units known as enumeration areas. The Avanza map provided additional information on the location and delineation process. The use of the map helped to streamline the data collection process and to ensure that the data were collected in a systematic and standardized manner. By clearly and accurately representing the study area, the enumerators could easily navigate the area and collect data from the designated sites. The data gathered using the ODK application was automatically uploaded to a secure server, stored, and processed for analysis. This ensured that the data were protected from loss and allowed for real-time monitoring of the data collection process.

Conducting data collection in urban households posed logistical and operational challenges. Unlike rural settings, urban populations are often more complex and unavailable around the house during the day when data collection was typically pursued. Therefore, navigating these diverse settings for data collection required well-trained enumerators and supervisors. Additionally, it requires multiple visits to find a respondent and arrange a particular interview time during the weekend or after working hours. The Addis-HDSS applied a robust field procedure to tackle these challenges. Proper and adequate training, coupled with supportive supervision, was provided to all field team members, and the study tools were tested in similar settings.

In conclusion, establishing an urban HDSS was accomplished because of the willingness of the population and the presence of a detailed digital map. The population structure provided helpful information on migration patterns into the city. The Addis-HDSS offers a research platform to inform public health practice and policy.
